# The rising potential of tooth germ-derived stem cells and the future of oral rehabilitation

**DOI:** 10.34172/japid.025.3960

**Published:** 2025-08-26

**Authors:** Adileh Shirmohammadi, Sina Ghertasi Oskouei

**Affiliations:** ^1^Dental and Periodontal Research Center, Tabriz University of Medical Sciences, Tabriz, Iran; ^2^Department of Periodontics, Faculty of Dentistry, Tabriz University of Medical Sciences, Tabriz, Iran; ^3^Section of Digital Dentistry, Faculty of Dentistry, Tabriz University of Medical Sciences, Tabriz, Iran

 It is a familiar scenario in dental practice: a patient seeks replacement for a missing tooth, and the conversation quickly turns to prosthetic options—whether fixed partial dentures, removable dentures, or dental implants. For decades, implant dentistry has been based on the ability to reconstruct bone volumes through guided bone regeneration, block grafts, and sinus floor augmentations, among other carefully refined surgical protocols. These techniques, in skilled hands, produce functional and esthetic success with high survival rates.^1^ Yet, transformative as they have been for modern practice, they focus on restoring the site for the implant, not the natural tooth itself.

 A different horizon is emerging from developmental biology: the capacity to grow a fully functional tooth, including the alveolar bone, periodontal ligament, and neurovascular structures, from the cells that would ordinarily produce it in nature—the tooth germ. Tooth germ-derived stem cells (TGSCs), harvested from unerupted third molars in their early formation stage, possess an intrinsic programming capable of synchronizing epithelial and mesenchymal contributions to tooth morphogenesis.^2^ This biological “blueprint” means that where these cells are appropriately seeded within a suitable 3D scaffold and exposed to the right signaling cues (such as Wnt/β-catenin, bone morphogenetic proteins, and fibroblast growth factors), they can direct the formation of a living organ rather than a single tissue.^3^

 In experimental in vivo settings, tooth germs transplanted into alveolar sites or bioengineered niches have led not only to crown and root dentin production but also to coordinated formation of surrounding bone.^4,5^ This phenomenon gives TGSC-based therapy a dual appeal: acting both as a regenerative solution for bone loss and as a pathway toward complete biological tooth replacement. In principle, if a reliable and safe method for patient-specific tooth germ regeneration is achieved, the future of oral rehabilitation could shift from implant integration to tooth regeneration (Figure 1).

**Figure 1:**
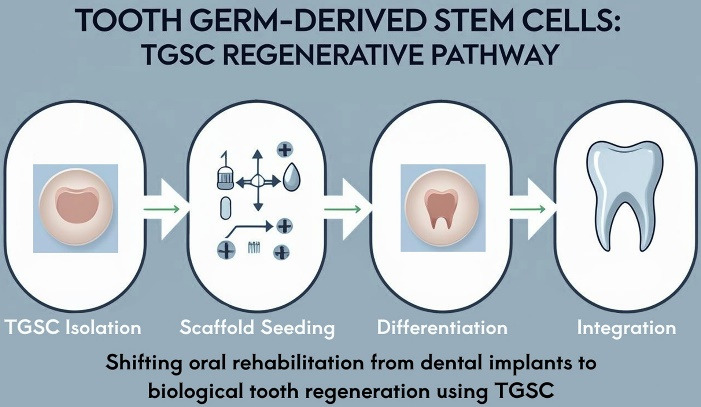
Schematic process of TGSC-based oral rehabilitation

 Recent studies have taken significant strides toward clinical plausibility. Bioengineered scaffolds designed to support TGSC survival have moved from simple collagen matrices to composite structures with controlled porosity, degradation kinetics, and mechanically tuned environments.^6,7^ In combination with microvascularization strategies—using angiogenic co-cultures or vascular growth factor delivery—the goal is to ensure the developing tooth bud is sustained throughout morphodifferentiation.^8^ Notably, clinical translation is advancing, with researchers affiliated with Kitano Hospital and Toregem BioPharma initiating human trials in October 2024 to assess an anti-USAG-1 antibody (TRG-035) that inhibits the USAG-1 protein, which suppresses tooth bud development, thereby reactivating latent tooth germ cells or their regenerative potential in edentulous areas.^9,10^ This approach aligns with TGSC research by leveraging the molecular pathways that govern tooth germ activation, offering a potential bridge to broader regenerative strategies.^11^

 For example, a preclinical study demonstrated that apical tooth germ cell-conditioned medium enhanced osteoblastic differentiation of periodontal ligament stem cells, with significantly increased alkaline phosphatase activity, mineralization, and expression of osteogenic markers like bone sialoprotein.^12^ This co-development is what sets TGSC-based regeneration apart from conventional graft-based augmentation: the bone is not a separate engineered element but a byproduct of the tooth’s natural development sequence. Furthermore, recent reviews underscore the promise of dental stem cells from tooth germs, such as dental follicle stem cells, in regenerating periodontal bony defects through osteogenic differentiation and angiogenic support, potentially diminishing the need for implants in cases with viable progenitor cells.^13^

 While the concept is compelling, several translational barriers remain. Isolation of viable TGSCs depends on accurate identification of an optimal developmental window—too early, and the cells may lack the resilience for in vitro handling; too late, and their differentiation pathway may have progressed beyond pluripotency.^9^ Expanding these cells in culture while preserving their inductive properties remains an active area of tissue engineering research.^10^ Another major challenge is predictability: natural tooth development is a tightly choreographed biological dance, and replicating it outside the embryonic jaw environment requires a deep understanding of morphogen gradients, temporal signaling, and mechanotransduction influences.^3^

 From a clinical standpoint, integration into dental workflows would require minimally invasive harvesting during routine oral surgery, biobank storage compatibility, and ready-to-use constructs for eventual implantation years later—raising questions of cost, regulation, and patient accessibility.^4^ Ethical and legal frameworks for organ-level regeneration in dentistry are still in their infancy, with approval pathways differing widely between jurisdictions. Safety considerations, particularly the avoidance of teratoma formation or ectopic mineralization, will demand rigorous long-term studies.

 Rather than framing TGSC-based regeneration as a competitor to existing implant dentistry, it is better viewed as a complementary advancement that avoids an unnecessary divide. In reality, these are sequential chapters in the evolution of oral rehabilitation. Implants and graft-based reconstruction represent the current gold standard—predictable, refined, and life-changing for millions worldwide. Tooth regeneration represents a likely next chapter, one in which biology and technology converge to restore an organ rather than replace it. The transition will likely be gradual, with TGSC-based protocols first applied in research settings or for patients where conventional therapies are contraindicated, and then expanding as techniques prove stable and reproducible.

 If current trends hold, the timeline from laboratory to early mainstream practice could be shortened by strategic collaboration between academic researchers, biotechnology firms, and clinicians keen to pilot these approaches in controlled settings. Standardization of stem cell sourcing, scalability of scaffold manufacturing, and regulatory clarity will accelerate translation. The long-term vision is compelling: an everyday dental visit where replacing a lost tooth could mean replanting a living bud that grows into a fully integrated tooth and supporting bone over months, restoring not just morphology and occlusion but the neurovascular and periodontal integration of a natural dentition.

 This would redefine oral rehabilitation—not merely reconstructing the jaw, but regenerating its living architecture. It is a goal that, if realized, will rewrite dental treatment planning for generations to come.

## Competing Interests

 Currently, Adileh Shirmohammadi is the Editor-in-Chief of the *Journal of Advanced Periodontology & Implant Dentistry* (JAPID), and Sina Ghertasi Oskouei serves as an Associate Editor for JAPID. The authors declare no other competing interests concerning authorship and/or publication of this article.
